# Modulation of HIV-1 Gag NC/p1 cleavage efficiency affects protease inhibitor resistance and viral replicative capacity

**DOI:** 10.1186/1742-4690-9-29

**Published:** 2012-04-01

**Authors:** Noortje M van Maarseveen, Dan Andersson, Martin Lepšík, Axel Fun, Pauline J Schipper, Dorien de Jong, Charles AB Boucher, Monique Nijhuis

**Affiliations:** 1 Dept. of Medical Microbiology, Virology, University Medical Center Utrecht, Heidelberglaan 100 (HP G04.614), 3584 CX Utrecht, the Netherlands; 2 Gilead Sciences and IOCB Research Center Prague, Institute of Organic Chemistry and Biochemistry, v.v.i., Academy of Science of the Czech Republic, Flemingovo n.2, 166 10 Praha 6, Czech Republic; 3 Dept. of Virology, Erasmus Medical Center, Dr. Molewaterplein 50, 3015 GE Rotterdam, the Netherlands

**Keywords:** HIV-1, Protease, Resistance, Gag, Cleavage, Replicative capacity, NC/p1

## Abstract

**Background:**

Mutations in the substrate of HIV-1 protease, especially changes in the NC/p1 cleavage site, can directly contribute to protease inhibitor (PI) resistance and also compensate for defects in viral replicative capacity (RC) due to a drug resistant protease. These NC/p1 changes are known to enhance processing of the Gag protein. To investigate the capacity of HIV-1 to modulate Gag cleavage and its consequences for PI resistance and RC, we performed a detailed enzymatic and virological analysis using a set of PI resistant NC/p1 variants (HXB2^431V^, HXB2^436E+437T^, HXB2^437T ^and HXB2^437V^).

**Results:**

Here, we demonstrate that single NC/p1 mutants, which displayed only a slight increase in PI resistance did not show an obvious change in RC. In contrast, the double NC/p1 mutant, which displayed a clear increase in processing efficiency and PI resistance, demonstrated a clear reduction in RC. Cleavage analysis showed that a tridecameric NC/p1 peptide representing the double NC/p1 mutant was cleaved in two specific ways instead of one.

The observed decrease in RC for the double NC/p1 mutant (HXB2^436E+437T^) could (partially) be restored by either reversion of the 436E change or by acquisition of additional changes in the NC/p1 cleavage site at codon 435 or 438 as was revealed during *in vitro *evolution experiments. These changes not only restored RC but also reduced PI resistance levels. Furthermore these changes normalized Gag processing efficiency and obstructed the novel secondary cleavage site observed for the double NC/p1 mutant.

**Conclusions:**

The results of this study clearly demonstrate that HIV-1 can modulate Gag processing and thereby PI resistance. Distinct increases in Gag cleavage and PI resistance result in a reduced RC that can only be restored by amino acid changes in NC/p1 which reduce Gag processing to an optimal rate.

## Background

The Human Immunodeficiency Virus type-1 (HIV-1) protease (PR) is a crucial enzyme in the viral life cycle. Its activity is required for the generation of mature infectious virus particles through the highly regulated and ordered cleavage of the viral precursor Gag and GagPol polyproteins. The Gag polyprotein encodes the structural proteins of the virus, which include matrix (MA), capsid (CA), nucleocapsid (NC), p6, and two spacer peptides p2 and p1. The GagPol protein, which is formed after a ribosomal frameshift event with a frequency of 5-10%, encodes in addition to the structural proteins the three viral enzymes protease, reverse transcriptase, and integrase.

Since the HIV-1 PR plays such a crucial role in the viral life cycle, it has proven to be a good target for antiretroviral therapy, and the introduction of HIV protease inhibitors (PI) has been one of the key components in the success of highly active antiretroviral therapy (HAART). Unfortunately, virological failure has been observed and related to the development of PI resistant viruses [1-4]. The evolution of PI resistance has been characterized as a stepwise process in which amino acid changes in the substrate-binding pocket or at more distant sites in the viral PR are selected initially. These amino acid changes directly or indirectly reduce the affinity of the viral PR for the inhibitor, thereby causing PI resistance. These amino acid changes also affect the binding of the viral PR to its natural substrate, the Gag and GagPol polyproteins, and as a consequence many of these PI resistant variants display a reduced replicative capacity (RC) as compared to wild-type virus [5-8]. To compensate for a diminished PR activity and thus for a reduced RC, PI resistant viruses may select compensatory changes in the viral PR itself or in the substrate of the viral PR, the Gag polyprotein [8-15]. Within the Gag polyprotein, compensatory changes have frequently been observed in the C-terminal region, in particular in the NC/p1 and p1/p6 cleavage sites [8,10-13,15-17].

More recently, it has been shown that changes in the NC/p1 cleavage site not only act as compensatory mutations, but can also directly contribute to PI resistance. *In vitro *selection experiments with an experimental high-genetic barrier PI (RO033-4649) resulted in the selection of K436E and/or I437T/V at the P4'and P5' positions of the NC/p1 cleavage site in the absence of mutations in the viral protease [18]. A similar observation was made by De Meyer *et al. *who reported the emergence of viruses carrying mutations in the NC/p1 cleavage site preceding the selection of mutations in the viral PR during *in vitro *selection experiments with darunavir [19]. Furthermore, we demonstrated that these NC/p1 changes confer PI resistance by enhancing the processing of Gag [18]. In addition, it has been demonstrated that NC/p1 mutations can strongly contribute to PI resistance in the presence of resistance-associated mutations in the viral protease besides compensating for loss in viral replicative capacity and are associated with therapy failure in vivo [17,20,21].

In this study, we investigated the capacity of HIV-1 to modulate Gag cleavage and its consequences for PI resistance and replicative capacity by performing a detailed enzymatic and virological analysis using a set of PI resistant NC/p1 variants (HXB2^431V^, HXB2^436E+437T^, HXB2^437T ^and HXB2^437V^).

## Results

### Effect of NC/p1 cleavage site mutations on viral RC and Gag processing

**Figure 1:**
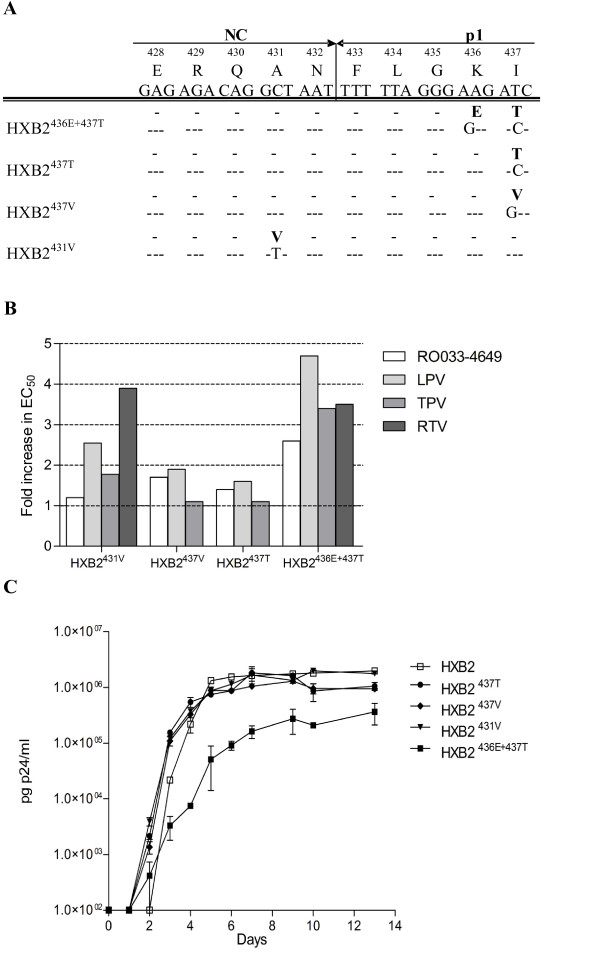
**Analysis of PI susceptibility and viral replicative capacity of NC/p1 cleavage site mutants**. (A) Sequences of the NC/p1 cleavage site mutants used in this study. Nucleotide changes and amino acid changes (bold) as compared to the wild-type virus HXB2 are indicated. (B) Analysis of drug susceptibility of the NC/p1 cleavage site mutants to RO033-4649, lopinavir (LPV), tipranavir (TPV) and ritonavir (RTV). Indicated are the fold changes in EC_50 _compared to wild-type. (C) Viral replication curves of the different NC/p1 cleavage site mutants (HXB2^436E+437T^, HXB2^437T^, HXB2^437V ^and HXB2^431V^) as compared to the wild-type HXB2. Error bars indicate the standard error of the mean.

A set of four HIV-1 HXB2 recombinant virus clones containing NC/p1 resistance mutations described in the literature and conferring different levels of PI resistance was generated: HXB2^431V^; HXB2^436E+437T^; HXB2^437T ^and HXB2^437V ^(Figure 1a &1b) [18]. The impact of these NC/p1 cleavage site changes on viral replicative capacity was investigated by performing viral replication curves in SupT1 cells. These experiments demonstrated that the single NC/p1 mutants (HXB2^431V^; HXB2^437T ^and HXB2^437V^), which displayed only a slight decrease in PI susceptibility, did not show an obvious change in replicative capacity, although small differences cannot be excluded (Figure 1c). In contrast, the double NC/p1 mutant (HXB2^436E+437T^), which displayed a clear decrease in PI susceptibility, also demonstrated a clear reduction in replicative capacity. We previously showed using quantitative Western blot analysis that this double NC/p1 mutant not only had an enhanced NC/p1 processing, but also an enhanced overall Gag processing compared to wild-type [18]. In addition to quantitative Western blotting, we also investigated NC/p1 cleavage by analysing hydrolysis of tridecameric peptides spanning the NC/p1 cleavage site by wild-type PR. Using an HPLC-based assay, in which peptide hydrolysis was quantified by peak area integration, it was observed that the NC/p1 peptide reflecting the original HXB2^436E+437T ^mutant (ERQANFLGETWPS) was cleaved approximately 2.4 fold more efficiently than the wild-type NC/p1 peptide (ERQANFLGKIWPS) (Table 1), which supports the results of the quantitative Western blot analysis.

**Table 1:** Normalized activity of NC/p1 peptide cleavages by HIV protease

Virus	Peptide	Normalized activity (± SD)
Wild-type (HXB2)	ERQANFLGKIWPS	100 ± 5.0
436E + 437 T	ERQANFLGETWPS	240 ± 3.6
+435R	ERQANFLRETWPS	210 ± 18.9
+438R	ERQANFLGETRPS	160 ± 6.7

Furthermore, it was observed that cleavage of the HXB2^436E+437T ^NC/p1 peptide gave rise to four product peaks after HPLC separation instead of two. Therefore, the four fragments were collected from preparative HPLC and examined by amino acid analysis; the results indicated that the cleavage products are the peptides ERQAN, ERQANF, LGETWPS, and FLGETWPS, indicating a potential secondary PR cleavage site in the HXB2^436E+437T ^NC/p1 peptide. LC-MS analysis confirmed that these peptides are indeed the correct cleavage products. To provide further evidence of their identity, the peptide products were synthesized and injected over the column. They yielded the same retention time as the peaks observed after enzymatic cleavage by HIV-1 PR.

### Evolution of NC/p1 mutants in absence of PI pressure and its impact on RC and PI resistance

To investigate the evolutionary potential of the NC/p1 mutants in the absence of PI pressure and to investigate if any reductions in RC could be restored, multiple *in vitro *evolution experiments were performed in SupT1 cells. After 10 passages, full Gag and PR were amplified and sequenced.

For the single NC/p1 mutants with only a slight increase in PI resistance and no obvious reduction in RC, no evolution was observed in Gag and PR (data not shown). The only exception was selection of a R429K change at the p4 position of the NC/p1 cleavage site in 1 out of 5 experiments starting with HXB2^431V^. When performing viral replication curves under modified assay conditions in which the amount of input 24 was lowered to discern subtle differences in RC, it was observed that the selection of the R429K slightly improved the RC of HXB2^431V ^(Additional file 1). In the control experiments, where HXB2 was cultured in the absence of PI, also no amino acid changes in Gag or PR were observed.

**Figure 2:**
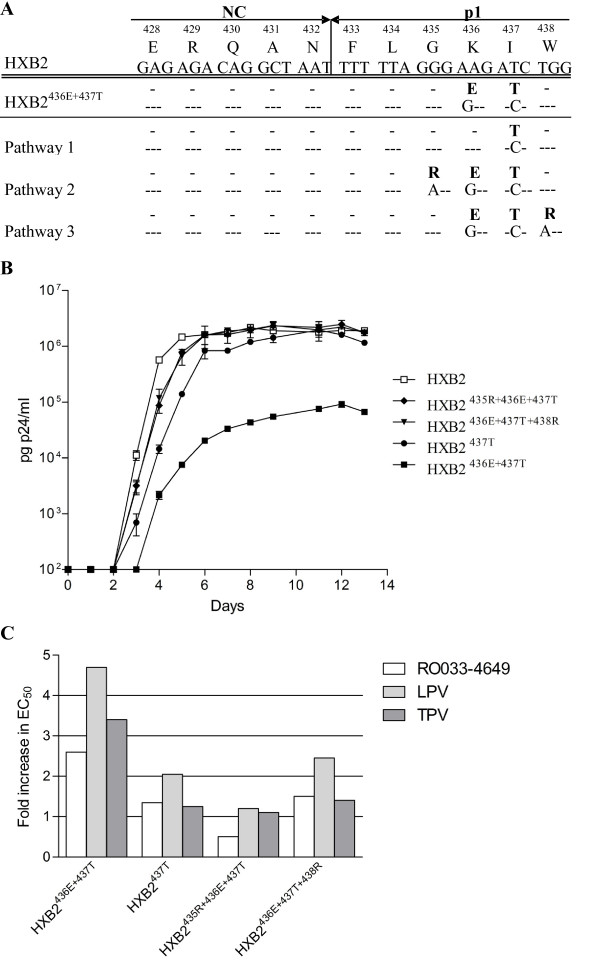
**Evolution of NC/p1 mutants in absence of PI pressure and its impact on RC and PI resistance**. (A) Representation of the evolutionary pathways observed during *in vitro *evolution experiments with HXB2^436E+437T^. Nucleotide changes and amino acid changes (bold) as compared to the wild-type virus HXB2 are indicated. (B) Viral replication curves of the different NC/p1 cleavage site mutants: HXB2^437T^, HXB2^435R+436E+437T ^and HXB2^436E+437T+438R ^as observed during *in vitro *evolution experiments of HXB2^436E+437T ^and compared to wild-type HXB2. Error bars indicate the standard error of the mean. (C) Representation of the fold increases in phenotypic drug resistance of the different NC/p1 cleavage site mutants: HXB2^437T^, HXB2^435R+436E+437T ^and HXB2^436E+437T+438R ^as observed during *in vitro *evolution experiments of HXB2^436E+437T ^and compared to wild-type HXB2. Drug susceptibility to the PI RO033-4649, lopinavir (LPV), and tipranavir (TPV) was determined in the multiple cycle MTT assay.

Remarkably, evolution experiments with the poorly replicating double NC/p1 mutant, revealed the selection of amino acid changes in/or near the NC/p1 cleavage site in all five experiments, while no other changes in Gag or PR were observed (Figure 2A). Three different evolutionary pathways could be observed for the double NC/p1 mutant. The first pathway, which was observed in 2 out of 5 experiments, was a reversion to wild-type of one of the NC/p1 cleavage site changes (E436K). The second pathway, observed in 2 out of 5 experiments, did not involve reversion but acquisition of an additional change at the P3' position of the NC/p1 cleavage site (G435R). Lastly, the third pathway, observed in 1 out of 5 experiments, resulted in the acquisition of an amino acid change at the more distant P6' position of the NC/p1 site (W438R).

Subsequently, we investigated the impact of these selected NC/p1 changes on viral RC. Therefore, the C-terminal part of Gag (p2-NC-p1-p6) and PR was cloned into HXB2. This resulted in the generation of three clones with the following changes compared to wild-type HXB2: HXB2^437T^, HXB2^435R+436E+437T ^and HXB2^436E+437T+438R^.

Viral replication curves in SupT1 cells demonstrated that all variants had an increased RC compared to the original HXB2^436E+437T ^mutant (Figure 2B). Both reversion to wild-type at codon 436 and the acquisition of an arginine at either codon 435 or 438 resulted in viruses with an RC comparable to wild-type HXB2, although small differences cannot be excluded.

To investigate the effect on PI sensitivity, we determined the susceptibility to the clinically used PI lopinavir and tipranavir and the experimental PI RO033-4649 (Figure 2C). Interestingly, all viruses demonstrated a reduction in PI resistance. This was most pronounced for the variant which selected the 435R change. This variant demonstrated such a reduction in PI resistance that it became fully PI susceptible and even hypersusceptible for RO033-4649. The other two variants with either the reversion at codon 436 or the acquisition of the 438R change still demonstrated some residual PI resistance.

### Evolution of NC/p1 mutants in absence of PI pressure and its impact on Gag cleavage

To determine whether the restoration of RC observed during the *in vitro *evolution experiments of the HXB2^436E+437T ^mutant affected Gag cleavage, in particular NC/p1 cleavage, we investigated the proteolytic processing of Gag both virologically and enzymatically. We previously showed using quantitative Western blot analysis that the HXB2^436E+437T ^mutant not only had an enhanced NC/p1 processing but also had an enhanced overall Gag processing compared to wild-type [18].

For virological analysis, 293 T cells were transfected with either a wild-type HXB2 or a NC/p1 mutant clone in the absence and presence of different concentrations of RO033-4649. Particle lysates were analyzed by quantitative Western blotting using antisera against NC, capsid (CA) or matrix (MA), and the relative amount of fully processed NC, CA or MA was compared to respectively all NC, CA or MA reactive products.

**Figure 3:**
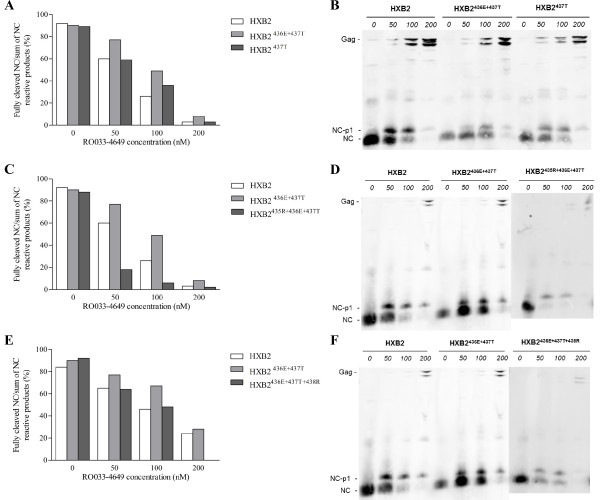
**Quantitative Western blot analysis of NC/p1 mutants using an anti-NC antibody**. Wild-type HXB2 and NC/p1 mutant clones used to transfect 293 T cells in the absence and presence of different concentrations of RO033-4649. Particle lysates were analyzed by quantitative Western blotting using an anti-NC antibody. Quantification of the NC-reactive signals and the original Western blots are presented in (A&B) for HXB2^437T^, in (C&D) for HXB2^436E+437T+438R ^and in (E&F) for HXB2^435R+436E+437T^.

**Figure 4:**
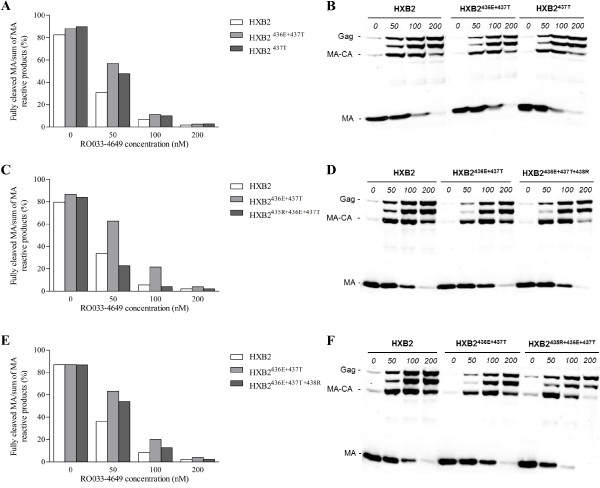
**Quantitative Western blot analysis of NC/p1 mutants using a MA antiserum**. Wild-type HXB2 and NC/p1 mutant clones used to transfect 293 T cells in the absence and presence of different concentrations of RO033-4649. Particle lysates were analyzed by quantitative Western blotting using a MA antiserum. Quantification of the MA-reactive signals and the original Western blots are presented in (A&B) for HXB2^437T^, in (C&D) for HXB2^436E+437T+438R ^and in (E&F) for HXB2^435R+436E+437T^.

When the NC/p1 processing of the *in vitro *evolution variants at suboptimal RO033-4649 concentrations of 50 and 100 nM was compared to the processing of the original HXB2^436E+437T ^mutant, it was observed that all selected variants restored NC/p1 processing (Figure 3). This was most pronounced for the acquisition of 435R, where processing levels were significantly reduced compared to the original double mutant and even wild-type (Figure 3E). Similar findings were obtained analysing overall Gag processing using antisera against MA and CA (Figure 4; CA data are shown in Additional file 2).

In addition to quantitative Western blotting, we also investigated NC/p1 cleavage by analysing hydrolysis of mutant tridecameric peptides spanning the NC/p1 cleavage site by wild-type PR. Using an HPLC-based assay, it was observed that the NC/p1 peptide reflecting the original HXB2^436E+437T ^mutant (ERQANFLGETWPS) was cleaved approximately 2.4 fold more efficiently than the wild-type NC/p1 peptide (ERQANFLGKIWPS) (Table 1) and gave rise to four product peaks after HPLC separation instead of two, indicating a novel secondary cleavage site.

When the cleavage of the *in vitro *evolution variants was compared to the cleavage of the original HXB2^436E+437T ^NC/p1 peptide, it was observed that acquisition of the 438R (ERQANFLGETRPS) significantly returned cleavage towards wild-type levels (Table 1). Acquisition of the 435R (ERQANFLRETWPS) also reduced cleavage, although this reduction was not significant. Furthermore, it was observed that both peptides gave rise to only two product peaks during HPLC analysis. This suggests that the secondary cleavage site observed for the HXB2^436E+437T ^NC/p1 peptide was obscured due to the acquisition of either the 435R or the 438R change.

### Molecular modeling and computation of HIV-1 protease-substrate binding

To investigate whether the changes in the NC/p1 substrate observed during the evolution experiments affected its binding to the viral PR (i.e. K_m _effect), molecular modeling was performed. Complexes of wild-type PR and seven tridecapeptide substrate variants were modeled and simulated using a 1-ns molecular dynamics run. The overall structure of the PR/substrate complexes was stable with a root-mean-square deviation (RMSD) of the PR backbone of 0.8 - 1.5 Å with respect to the starting crystal structure (PDB code: 2FNS). Further analyses were done using the last 500 ps of the simulations to allow for longer equilibration.

Positional fluctuations of substrate residues showed a pronounced difference between the P3-P3' residues within the active site on the one hand and the residues outside the PR cavity on the other. The former group had a smaller calculated average ADP (atomic displacement parameter, formerly B-factor) of 44.9 ± 15.4 Å^2^, whereas the latter had a higher average ADP of 103.0 ± 50.5 Å^2 ^(Additional file 3). The former value could be compared with the average experimental ADPs of P3-P3' residues of 67.5 ± 0.9 Å^2 ^from the original crystal structure.

These differences in the flexibility between P3-P3' and the flanking substrate residues are reflected in the spread of the energy contributions of substrate residues to the PR-substrate interaction, which is larger for the whole peptide (16.6 kcal/mol) than for the better-localized P3-P3' residues only (11.6 kcal/mol) (Additional file 4). Due to the difficulties in comparing the energies of the structurally diverse flanking substrate residues, we have focused on their effect upon the binding of P3-P3' residues. Overall, with the exception of A431V, the energy values are within a small range of 3.4 kcal/mol. Given the systematic errors in the computed values of a few kilocalories per mole due to approximations in the molecular model and limited sampling, we have interpreted the results with caution. We thus observe the strongest binding between the HIV-1 PR and the P2 mutated substrate (A431V), whereas considerably weaker binding occurs with all the other peptides, including wild-type. However, due to the small differences in their interaction energy values, it is difficult to discern differences between the NC/p1 variants that emerged from the evolution experiments and the original HXB2^436E+437T ^variant.

## Discussion

In this study, we investigated the capacity of HIV-1 to modulate Gag cleavage and its consequences for PI resistance and RC by using a set of PI resistant NC/p1 variants (HXB2^436E+437T^, HXB2^437T^, HXB2^437V ^and HXB2^431V^). The 431 V and 437 V changes have been observed frequently in relation to PI resistance both *in vitro *and *in vivo*, whereas the 436E + 437 T changes as well as the 437 T change have only been observed *in vitro *so far [18,22-24].

HIV-1 Gag processing is highly regulated and results in the ordered generation of mature viral proteins. During the processing of Gag, cleavage of NC/p1 is considered to be among the last and slowest events [25]. This slow processing rate of NC/p1 seems to be the consequence of a suboptimal amino acid sequence, resulting in a suboptimal conformation of the NC/p1 site for binding to the HIV-1 PR [26], as we have also shown in this study. Site-directed mutagenesis experiments have shown that the processing rate of NC/p1 can be accelerated by certain amino acid changes at codon 431 and 432, the P2 and P1 positions of this cleavage site [27]. We previously demonstrated that under PI selective pressure, the virus may select amino acid changes in NC/p1 at codons 431, 436 and 437 (the P2, P4' and/or P5' positions) which cause PI resistance due to an enhanced processing of not only NC/p1, but also Gag [18].

This study shows that viral variants with single NC/p1 resistance mutations, A431V, I437V or I437T with only low-level PI resistance comparable to resistance levels observed for single resistance mutations in PR, do not display an obvious reduction in RC. This is in line with a study from Pettit *et al*, demonstrating that site-directed mutants displaying an enhanced processing rate of NC/p1 due to amino acid changes at the P1 position of the NC/p1 site showed significant levels of infectivity. In contrast, the viral variant with two amino acid changes at the NC/p1 site, 436E and 437 T, which displayed more pronounced levels of PI resistance, also demonstrated a clear reduction in RC. These data suggest that processing of NC/p1 can be enhanced to a certain level without major consequences for the RC, but that above this level RC is severely affected.

This observation is also supported by the *in vitro *evolution experiments in which we investigated if the reduced RC of the double NC/p1 mutant could be restored. In absence of inhibitor, the reduced viral RC of drug-resistant variants can be (partially) restored by loss of the drug resistance conferring amino acid changes (E436K; reversion to wild-type) or acquisition of compensatory mutations (G435R or W438R; persistence of drug resistance mutations) [28]. Despite the fact that there are constraints to the selection of amino acid changes in the NC/p1 site, because the underlying coding sequence also regulates the Gag/GagPol ribosomal frameshift, only changes in the NC/p1 site were selected. This implies that the reduced RC of the double NC/p1 variant can only be restored by normalizing NC/p1 cleavage through selection of changes in this particular site. Indeed, we demonstrated with both quantitative immunoblotting and peptide cleavage analysis that all observed changes reduced NC/p1 and Gag processing. In parallel, PI susceptibility increased with the largest increase observed for the compensatory change G435R, which even resulted in hypersusceptibility to RO033-4649. One could speculate that when an enhanced Gag processing leads to PI resistance a reduction in processing may lead to PI hypersusceptibility.

Cleavage analysis of the original HXB2^436E+437T ^variant showed that the model peptide ERQANFLGETWPS can be cleaved in two specific ways, ERQAN-FLGETWPS and ERQANF-LGETWPS, to yield the four products ERQAN, ERQANF, LGETWPS, and FLGETWPS. This shift to ERQANF-LGETWPS represents a novel cleavage site for NC/p1, which partly (P2-P1*P1') mimics the p1-p6 cleavage site PGNF*LQSR. This secondary cleavage site may to some extent explain the higher activity observed for the HXB2^436E+437T ^peptide compared to the wild-type HXB2 peptide. Unfortunately, it is not possible to determine if the change in sequence or the secondary cleavage site is responsible for the higher activity. Selection of either the 435R or 438R change during the evolution experiments seems to obstruct this novel cleavage site, and in the case of the 438R it results in a significant reduction in cleavage activity. Further analysis of the Gag cleavage products *in vivo *will be necessary to analyze the possible role of this putative cleavage site in the maturation of HIV-1 particles.

We have attempted to explain the molecular mechanism underlying the selection of mutations during the evolution experiments and their effects on Gag processing, RC and PI susceptibility. Using molecular dynamics simulations of seven PR-substrate complexes in which we focused on possible effects on K_m_, we found that the P4'-P8' region of the NC/p1 substrate was very flexible, which is in line with the lack of corresponding electron densities in the X-ray structure of the corresponding complex [29].

The interaction energy calculations showed that the NC/p1 substrate harbouring the A431V change binds strongest to the PR, whereas the other substrates were bound more weakly. Due to the small energy differences, which are similar in magnitude to the systematic errors, these could not be sorted further. Our finding that the substrate with the A431V mutation binds more tightly to the PR than the wild-type NC/p1 substrate is supported by previous molecular modeling and may in part explain the higher K_m_/k_cat _value observed for the cleavage of the mutated peptide [18,30]. The very similar interaction energies of the P3-P3' moieties of the other substrates of the analyzed series could be caused by the fact that the effect of the flanking P4'-P8' residues on the binding of the P3-P3' residues is very small. One should also bear in mind that apart from the effect on K_m_, the mutations may influence the barriers of the cleavage reactions and affect k_cat _as well. In summary, a molecular description of the mechanims underlying the effect of substrate mutations on Gag processing is currently at the limit or even beyond of today's computational tools.

## Conclusions

In conclusion, the results of this study indicate that HIV-1 can modulate NC/p1 cleavage and thereby PI susceptibility. We show that processing of NC/p1 can be enhanced to a certain level without major consequences for RC, but that above this level RC is severely affected. In addition, we demonstrate that once RC is reduced, the virus may modulate its NC/p1 sequence by selecting additional changes that restore Gag processing, PI susceptibility and RC.

## Methods

### Cells

SupT1 and MT-2 cells were maintained in RPMI 1640 medium with L-glutamine (BioWhittaker, Verviers, Belgium) supplemented with 10% fetal bovine serum (FBS; Gibco, Breda, The Netherlands) and 10 μg/ml gentamicin (Gibco). 293 T cells were maintained in Dulbecco's modified Eagle's medium (BioWhittaker) supplemented with 10% FBS and 10 μg/ml gentamicin. All cells were passaged twice weekly.

### Construction of NC/p1 HIV-1 molecular clones

PI resistant viruses harboring NC/p1 changes were generated during *in vitro *selection experiments using the experimental PI RO033-4649 or the common PI ritonavir [18]. Using the viruses obtained from the *in vitro *selections, recombinant viruses were generated as previously described [31]. This resulted in the generation of four different clones containing the following changes compared to wild-type HXB2: HXB2^431V ^(A431V), HXB2^436E+437T ^(K436E + I437T), HXB2^437T ^(I437T + A15T in p6^pol^), and HXB2^437V ^(I437V) (Figure 1A).

### Generation of recombinant viruses

To generate recombinant viruses, NC/p1 molecular clones were transfected in 293 T cells. For this, 5-6 × 10^6 ^293 T cells were seeded the day prior to transfection to achieve 90-95% confluence on the day of transfection. For transfection, 10 μg of plasmid DNA and Lipofectamine 2000 (Invitrogen) were used according to the manufacturer's instructions. Two days after transfection, recombinant virus was harvested.

### Viral replication experiments

To investigate the RC of the NC/p1 mutants, viral replication curves were generated. For each 293 T cell recombinant virus batch the amount of p24 was determined by ELISA (AMPAK™, DAKO, Cambridgeshire, UK) [31,32]. Viral replication experiments were performed by infecting 2.0 × 10^6 ^SupT1 cells with 100 ng of p24 of each recombinant virus batch. After two hours of incubation, the cells were washed twice with RPMI 1640 medium with L-glutamine (Cambrex, Verviers, Belgium) and resuspended in 10 ml culture medium (RPMI 1640 medium with L-glutamine supplemented with 10% FCS (Gibco, Breda, The Netherlands) and gentamicin (10 μg/ml, Invitrogen, Breda, The Netherlands). Cultures were maintained for 13 days and at day 0-7, 9, 10, and 13, two times 150 μl of cell-free viral supernatant was taken for p24 analysis.

### *In vitro *evolution experiments

*In vitro *evolution experiments were performed in five-fold for all NC/p1 mutants. For this SupT1 cells (2.0 × 10^6^) were infected with 250 μl recombinant virus in an initial volume of 1 ml culture medium. After 1 h incubation at 37°C, 9 ml of culture medium were added. Cultures were replenished with fresh culture medium twice weekly. When full-blown cytopathogenic effects (CPE) were observed, the virus was harvested, and approximately 12.5 - 250 μl were used to perform a new passage. After 10 passages, all Gag cleavage sites and the complete C-terminus of Gag (p2-NC-p1-p6) and protease were amplified from viral RNA and sequenced, as described previously [18,31]. Dominant amino acid changes compared to the original NC/p1 mutants were scored.

### Quantitative Western blot analysis

Recombinant NC/p1 HIV-1 molecular clones were used to transfect 293 T cells in the absence and presence of various concentrations of RO033-4649 using Lipofectamine 2000 (Invitrogen). After 48 h, culture medium was harvested, and virus particles were collected from cleared media by centrifugation at 17.000 rpm for 1 h at 4°C (Biofuge, fixed-angle rotor #3332, Heraeus, Germany). For Western blot analysis, viral supernatant was boiled in SDS sample buffer, separated by 12% SDS-PAGE for MA and CA analysis or by 16.5% Ready Gel Tris-Tricine gel (Bio-Rad, Veenendaal, The Netherlands) for NC analysis and transferred to an Immobilon-FL membrane (Millipore B.V., The Netherlands). The membrane was first probed with either an anti-NC antibody obtained from Dr. Jeff Lifson from the AIDS and Cancer Virus Program (SAIC Frederick, National Cancer Institute at Frederick, USA) or with HIV-1_SF2 _p24 antiserum, obtained through the AIDS Research and Reference Reagent Program (Division of AIDS, NIAID, NIH) or with an MA antiserum obtained from Prof. H.-G. Kräusslich from the University of Heidelberg (Germany) after which the membrane was probed with Alexa Fluor 680 goat anti-rabbit IgG (Invitrogen). Subsequently, quantitative Western blot analysis was performed using the Odyssey Infrared Imaging System as recommended by the manufacturer (LI-COR Biosciences, Munich, Germany).

### Drug susceptibility analysis

To determine drug susceptibility of the recombinant viruses, the infectious virus titre (TCID_50_) was determined using end-point dilutions in MT-2 cells. Subsequently, drug susceptibility of these viruses was determined in duplicate using the multiple cycle MTT assay [33].

### Hydrolysis of NC/p1 peptides

Peptides mimicking the wild-type and mutated cleavage site of NC/p1 were synthesized by the Peptide Synthesis Unit at the Institute of Organic Chemistry and Biochemistry, Academy of Sciences, Prague, Czech Republic. Cleavage of these peptides by wild-type HIV-1 PR was assayed at 37°C in 100 mM acetate buffer, pH 4.7, and 100 mM NaCl, using 500 nM HIV-1 PR (expressed and purified as previously described) and 200 μM substrate [34-36]. Reactions were allowed to proceed for 20-60 min, which corresponds to less than 15% substrate turnover under these conditions, and were stopped by addition of formic acid to a final concentration of 2%. Reactions were centrifuged at 13000 *g *for 10 min and then analyzed by HPLC. An Agilent 1200 HPLC system with an Agilent Zorbax SB-C18 RRHT threaded column thermostated at 60°C was used for separation of reaction mixtures. A gradient of 2% to 33% acetonitrile supplemented with 0.1% formic acid was used as a mobile phase. Separation was performed using a flow rate of 1.3 ml/min, and the duration of the gradient (6-16 min) was adjusted depending on the peptide so as to achieve separation between substrate and products. Substrate hydrolysis was quantified by integration of the substrate peaks and normalized with respect to wild-type, which was set to 100.

### Determination of NC/p1 peptide hydrolysis products

An Agilent preparative 1200 system, with an Agilent Zorbax SB-C18 RRHT threaded column, was used to separate and collect the NC/p1 peptide hydrolysis products. The identity of these hydrolysis products was determined by amino acid analysis and independently confirmed by LC-MS (hybrid FTMS mass spectrometer LTQ Orbitrap XL (Thermo Scientific) coupled with UHPLC system RHEOS Allegro (Flux Instruments) with ACQUITY UPLC BEH C18 (1.7 um × 2.1 mm × 150 mm) column (Waters)). Samples were measured in electro spray positive mode.

The peptides corresponding to the cleavage products identified by these analyses were synthesized and used as retention markers in the HPLC-based assay described above.

### Molecular modeling and simulations

The effect of NC/p1 substrate mutations on PR-substrate binding (K_m _effect) was investigated in seven PR/substrate complexes (wild-type NC/p1, HXB2^437T^, HXB2^437V^, HXB2^436E+437T^, HXB2^436E+437T+438R^, HXB2^435R+436E+437T^, and HXB2^431V^) using molecular modeling and simulations. The starting geometry was taken from the crystal structure of an inactive (D25N) wild-type HIV-1 PR complexed with the NC-p1 substrate peptide (PDB code: 2FNS)[29]. For substrate residues outside the well-defined S3-S3' PR pockets, no experimental electron densities had been observed. We thus modeled the flanking amino acids on both unprimed and primed sides to yield the tridecapeptides studied experimentally. The residues were added so as not to clash with van der Waals surface of PR visualized with Insight II package (Insight II; Accelrys Software Inc., 2000) nor with the crystallographic waters that were all included in the model.

The seven PR and NC/p1 complexes were set up for calculations, relaxed and solvated in a box of water molecules (details can be found in Additional file 5). Molecular dynamics simulations consisted of three steps: i) gradual warming to 300 K over 50 ps, ii) equilibration at 300 K for 200 ps and iii) production run at 300 K for 1 ns. Snapshots of the trajectory were saved every 1 ps.

The binding energies between the PR and the substrates were calculated using the molecular mechanics-generalized Born/surface area (MM-GBSA) methodology using the MM-PBSA and SANDER modules of AMBER 8 (D.A. Case, T.A. Darden, T.E. Cheatham, III, C.L. Simmerling, J. Wang, R.E. Duke, R. Luo, K.M. Merz, B. Wang, D.A. Pearlman, M. Crowley, S. Brozell, V. Tsui, H. Gohlke, J. Mongan, V. Hornak, G. Cui, P. Beroza, C. Schafmeister, J.W. Caldwell, W.S. Ross, and P.A. Kollman (2004), AMBER 8, University of California, San Francisco)[37]. Energies of the complex, PR and substrate (E_cplx_, E_PR_, and E_substr_, respectively) were evaluated for 100 snapshots (every 5 ps) from the second half of the trajectory. Their differences (*i.e*. E_cplx _- E_PR _- E_substr_) were averaged to yield the total PR-substrate interaction energies. These were further decomposed to contributions from individual amino acids.

## Competing interests

The authors declare that they have no competing interests.

## Authors' contributions

NMM, CAB and MN conceived and designed the study. NMM, DA, ML, AF, PJS and TJ performed the experiments and analyzed the data. NMM, DA, ML and MN wrote the paper with helpful comments of AF, PJS, TJ and CAB. All authors have read and approved the final version of the manuscript.

## Supplementary Material

Additional file 1 **Viral replication curves of HXB2^431V ^and HXB2^429K+431V ^as observed during *in vitro *evolution experiments of HXB2^431V^**. Click here for file

Additional file 2 **Quantitative Western blot analysis of NC/p1 mutants using a CA antiserum**. Click here for file

Additional file 3 **Residue positional fluctuations of substrates in complex with PR during molecular dynamics simulations**. Click here for file

Additional file 4 **Interaction energy between PR and substrate variants for tridecameric peptides and P3-P3' residues**. Click here for file

Additional file 5 **Molecular modeling and simulations of PR-substrate complexes**. Click here for file
