# Supplementary material for: Modulation of HIV-1 Gag NC/p1 cleavage efficiency affects protease inhibitor resistance and viral replicative capacity
Source: Retrovirology. 2012 Apr 1;9:29. doi: 10.1186/1742-4690-9-29 (PMC3349524; doi:10.1186/1742-4690-9-29)

**Additional file 1. Viral replication curves of HXB2^431V^ and HXB2^429K+431V^ as observed during in vitro evolution experiments of HXB2^431V^.**

Error bars indicate the standard error of the mean.


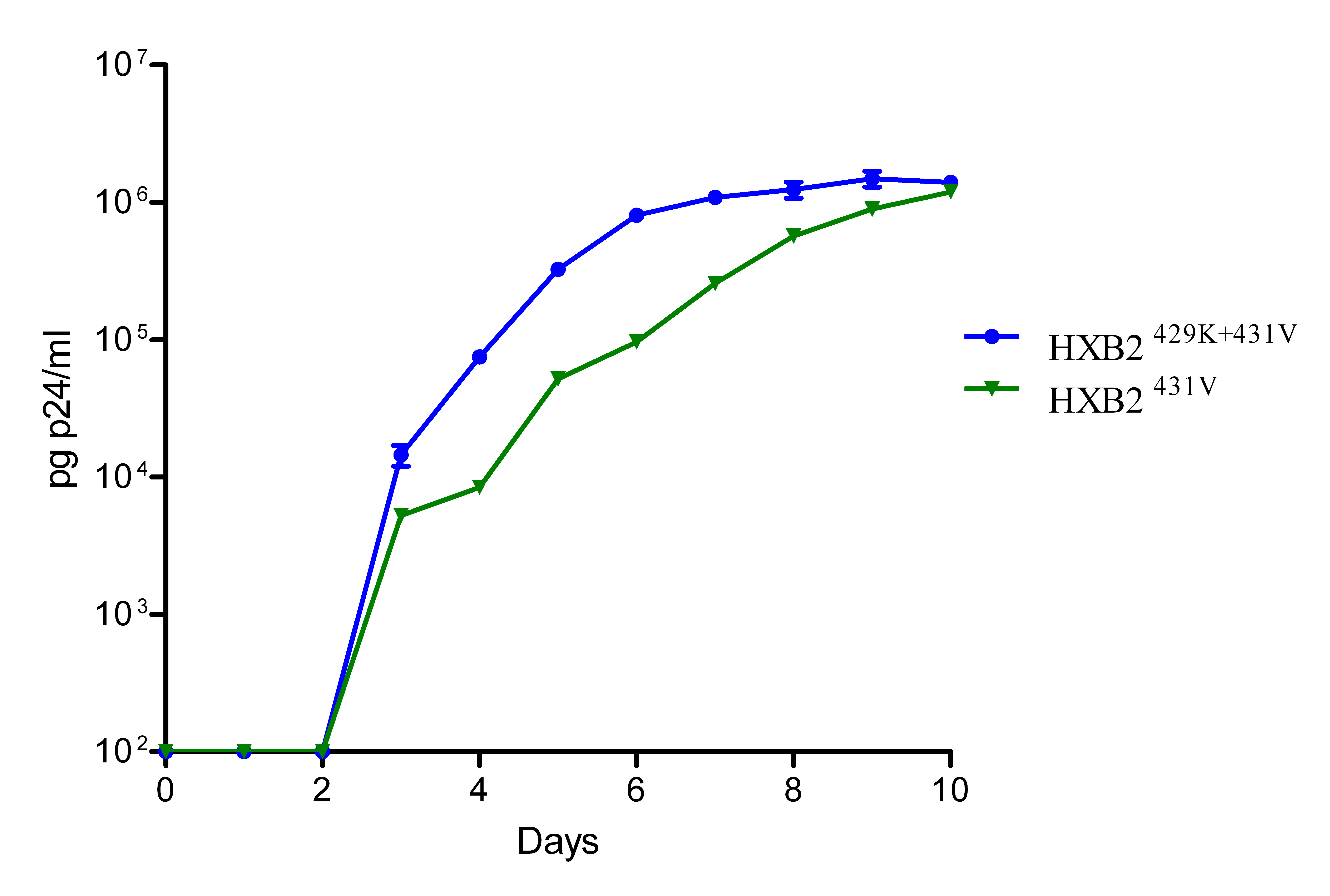

Supplement: Additional file 1 — Viral replication curves of HXB2431V and HXB2429K+431V as observed during in vitro evolution experiments of HXB2431V. [file 1742-4690-9-29-S1.DOC]
