# Supplementary material for: Modulation of HIV-1 Gag NC/p1 cleavage efficiency affects protease inhibitor resistance and viral replicative capacity
Source: Retrovirology. 2012 Apr 1;9:29. doi: 10.1186/1742-4690-9-29 (PMC3349524; doi:10.1186/1742-4690-9-29)
Supplement: Additional file 2 — Quantitative Western blot analysis of NC/p1 mutants using a CA antiserum. [file 1742-4690-9-29-S2.DOC]

**Additional file 2. Quantitative Western blot analysis of NC/p1 mutants using a CA antiserum.** Wild-type HXB2 and NC/p1 mutant clones were used to transfect 293T cells in the absence and presence of different concentrations of RO033-4649. Particle lysates were analyzed by quantitative Western blotting using a CA antiserum. Quantification of CA-reactive signals and the original Western blots are presented in (A & B) for HXB2^437T^, in (C&D) for HXB2^436E+437T+438R^ and in (E&F) for HXB2^435R+436E+437T^.

**A B**

**
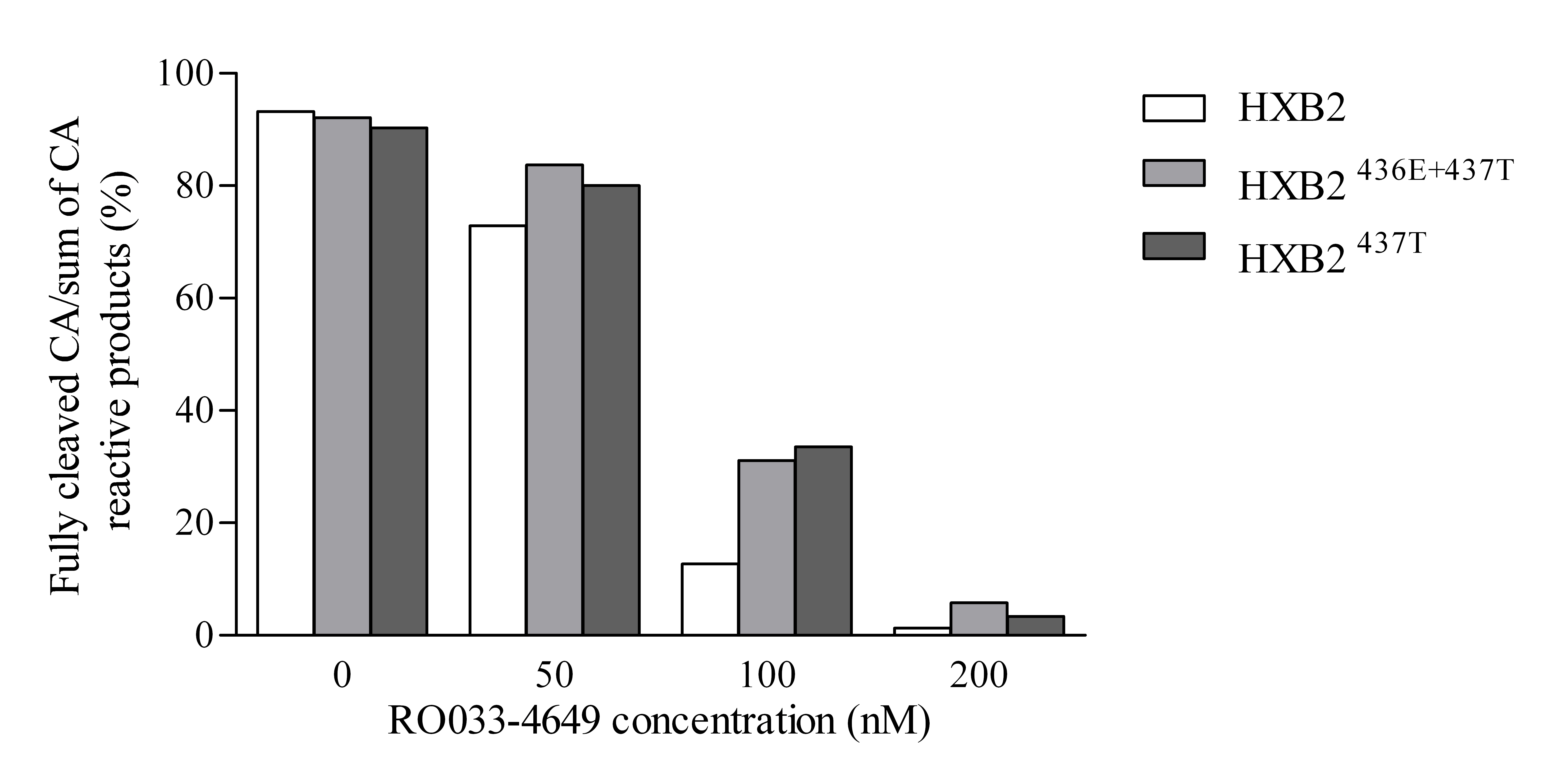
**

**C D**

**
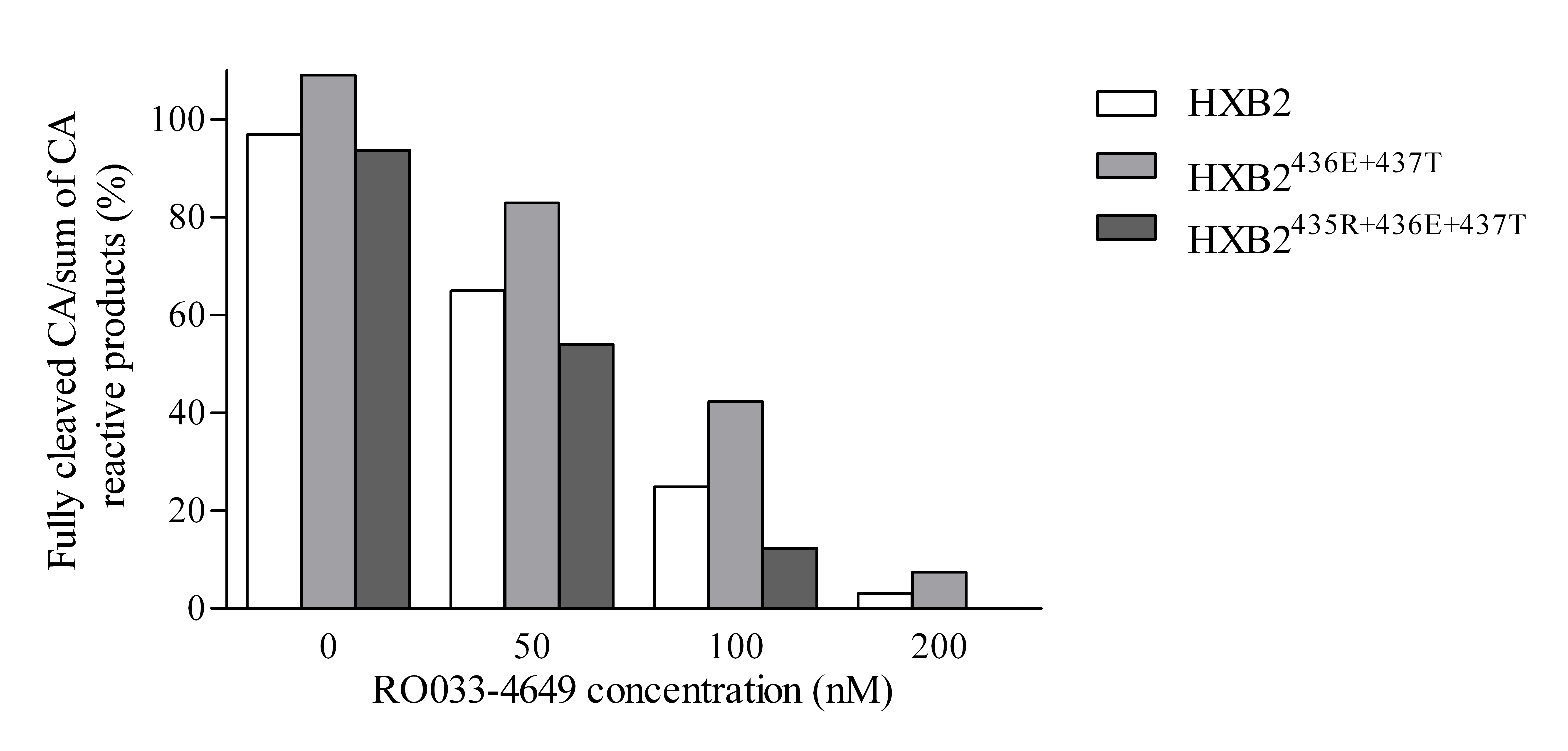
**

**E F**

**
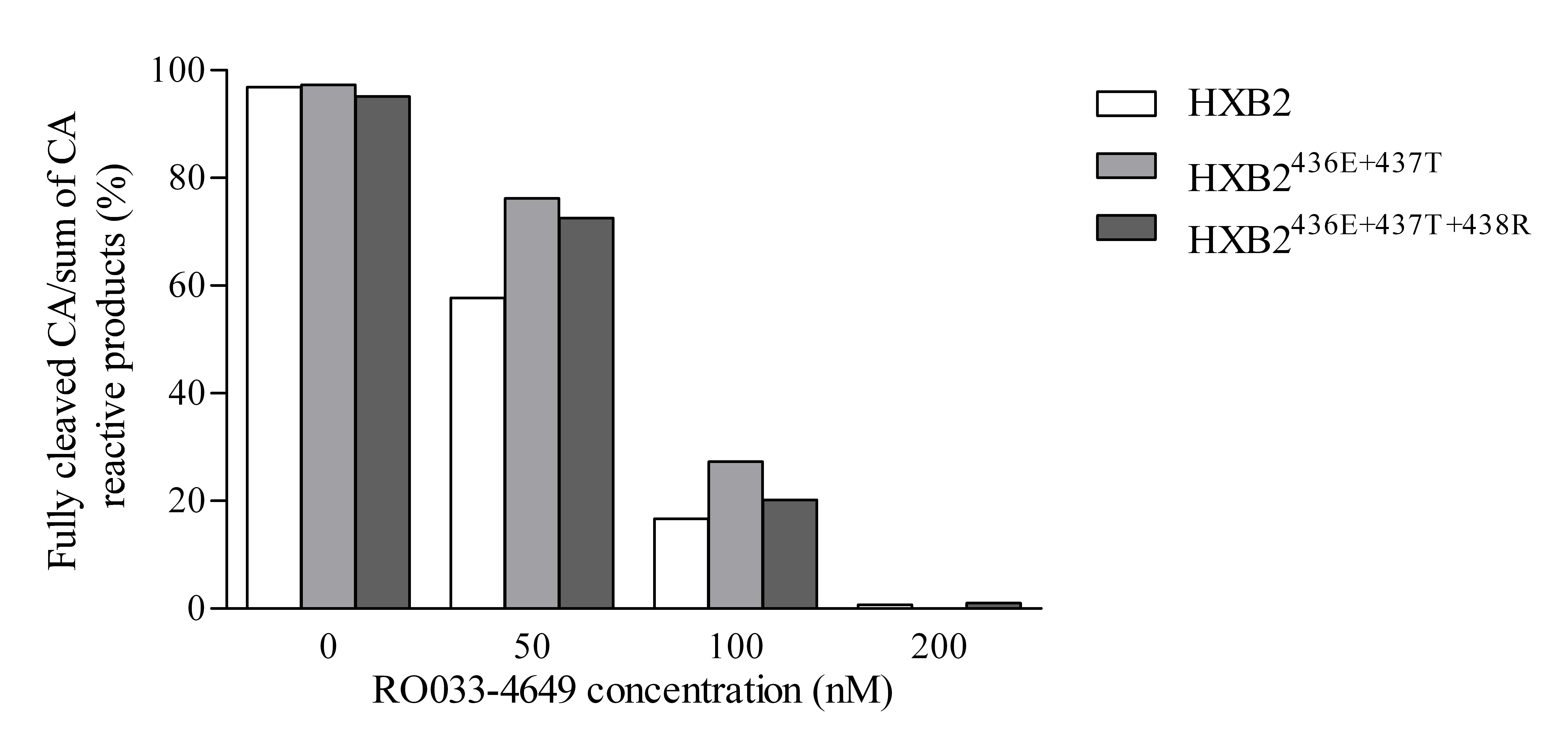
**
