# Supplementary material for: Modulation of HIV-1 Gag NC/p1 cleavage efficiency affects protease inhibitor resistance and viral replicative capacity
Source: Retrovirology. 2012 Apr 1;9:29. doi: 10.1186/1742-4690-9-29 (PMC3349524; doi:10.1186/1742-4690-9-29)
Supplement: Additional file 3 — Residue positional fluctuations of substrates in complex with PR during molecular dynamics simulations. [file 1742-4690-9-29-S3.DOC]

**Additional file 3. Residue positional fluctuations (ADPs, atomic displacement parameters, Å^2^) of substrates in complex with PR during molecular dynamics simulations.** Note the smaller calculated average ADPs of 44.9 ± 15.4 Å^2^ for the P3-P3‘ residues, in contrast with the higher average ADP of 103.0 ± 50.5 Å^2^ for the flanking residues.

*
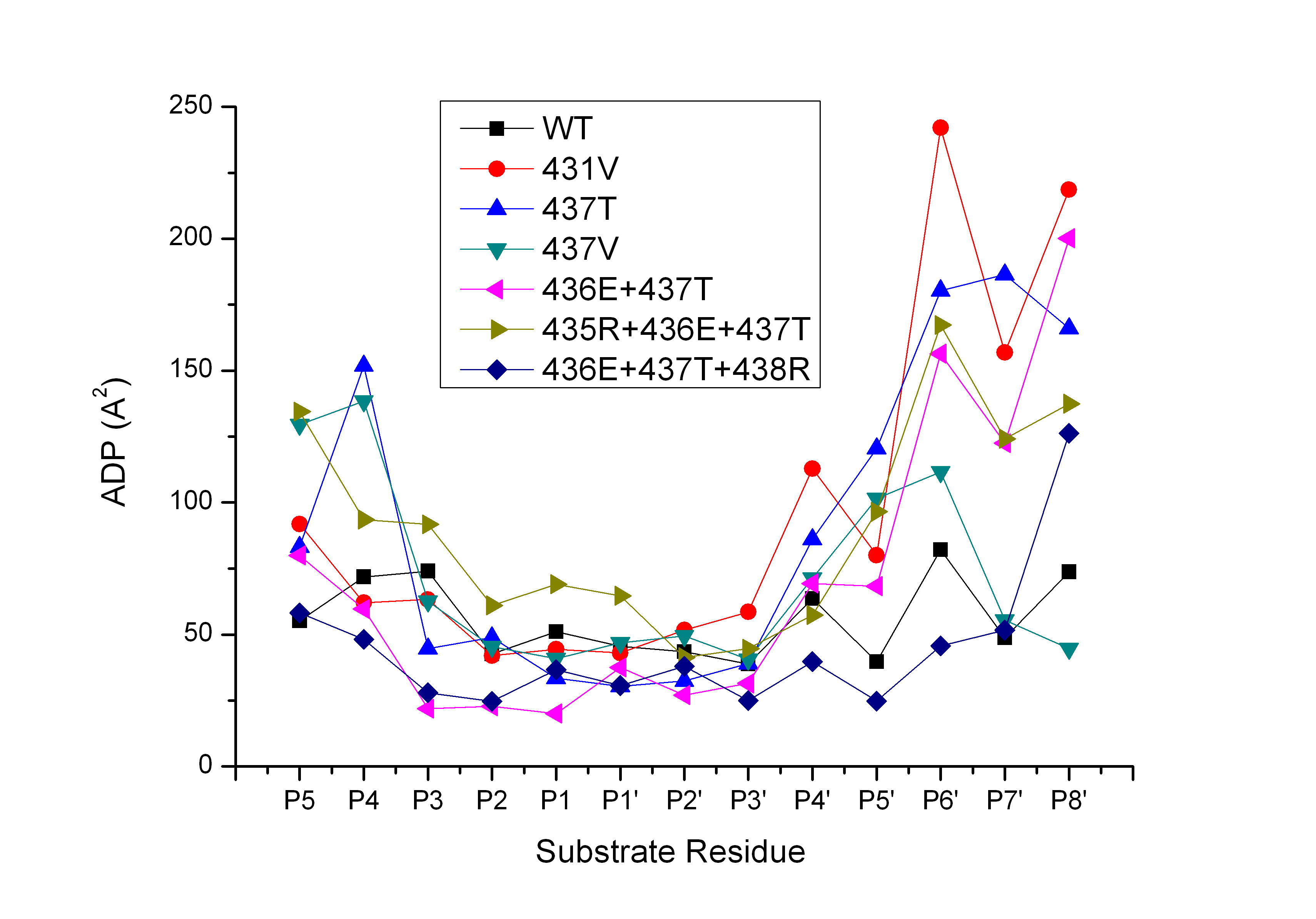
*
