# Supplementary material for: Modulation of HIV-1 Gag NC/p1 cleavage efficiency affects protease inhibitor resistance and viral replicative capacity
Source: Retrovirology. 2012 Apr 1;9:29. doi: 10.1186/1742-4690-9-29 (PMC3349524; doi:10.1186/1742-4690-9-29)

**Additional file 4. Interaction energy (kcal/mol) between PR and substrate variants for**

**(A) tridecameric peptides and (B) P3-P3’ residues.**

**A**


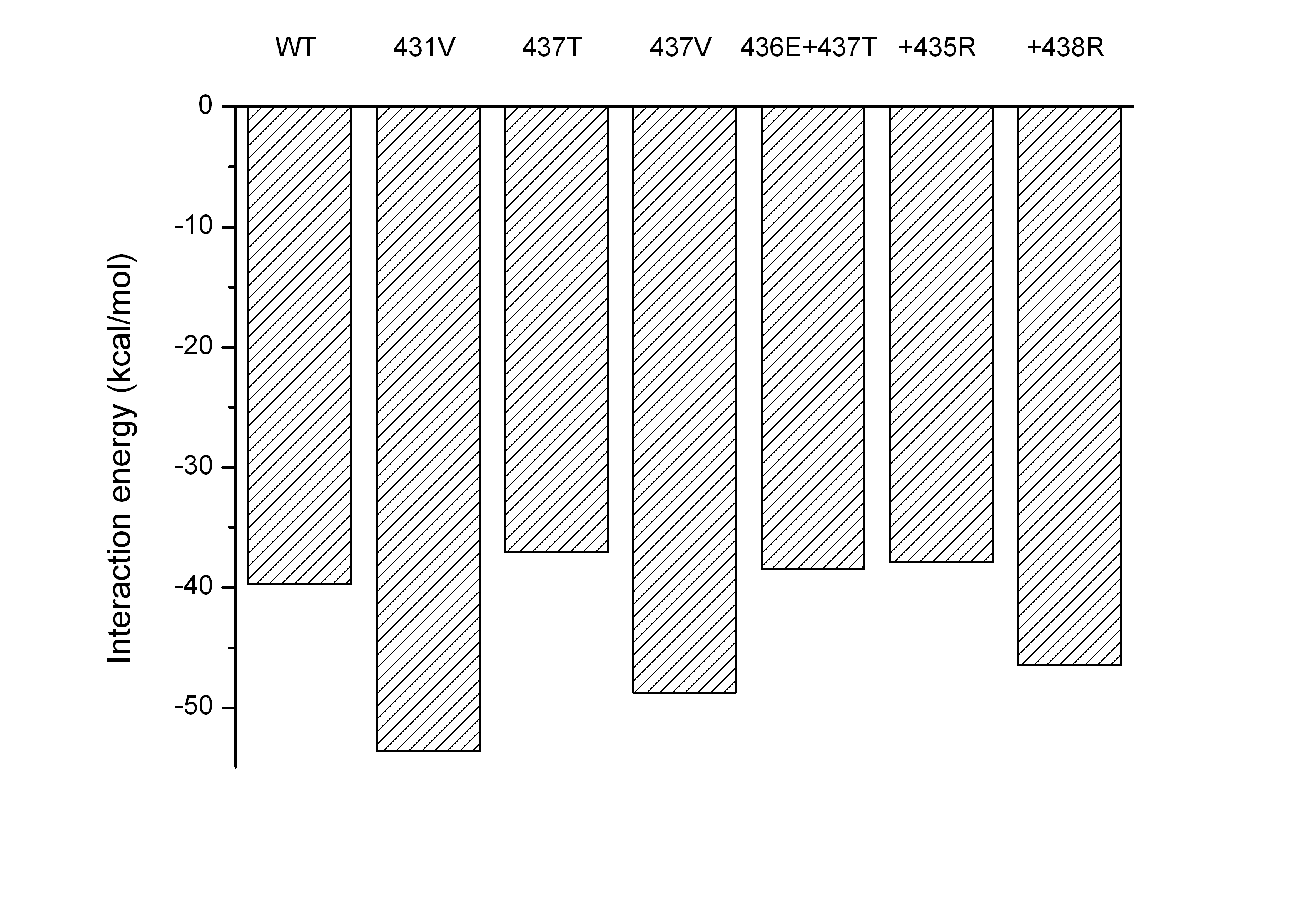


**B**


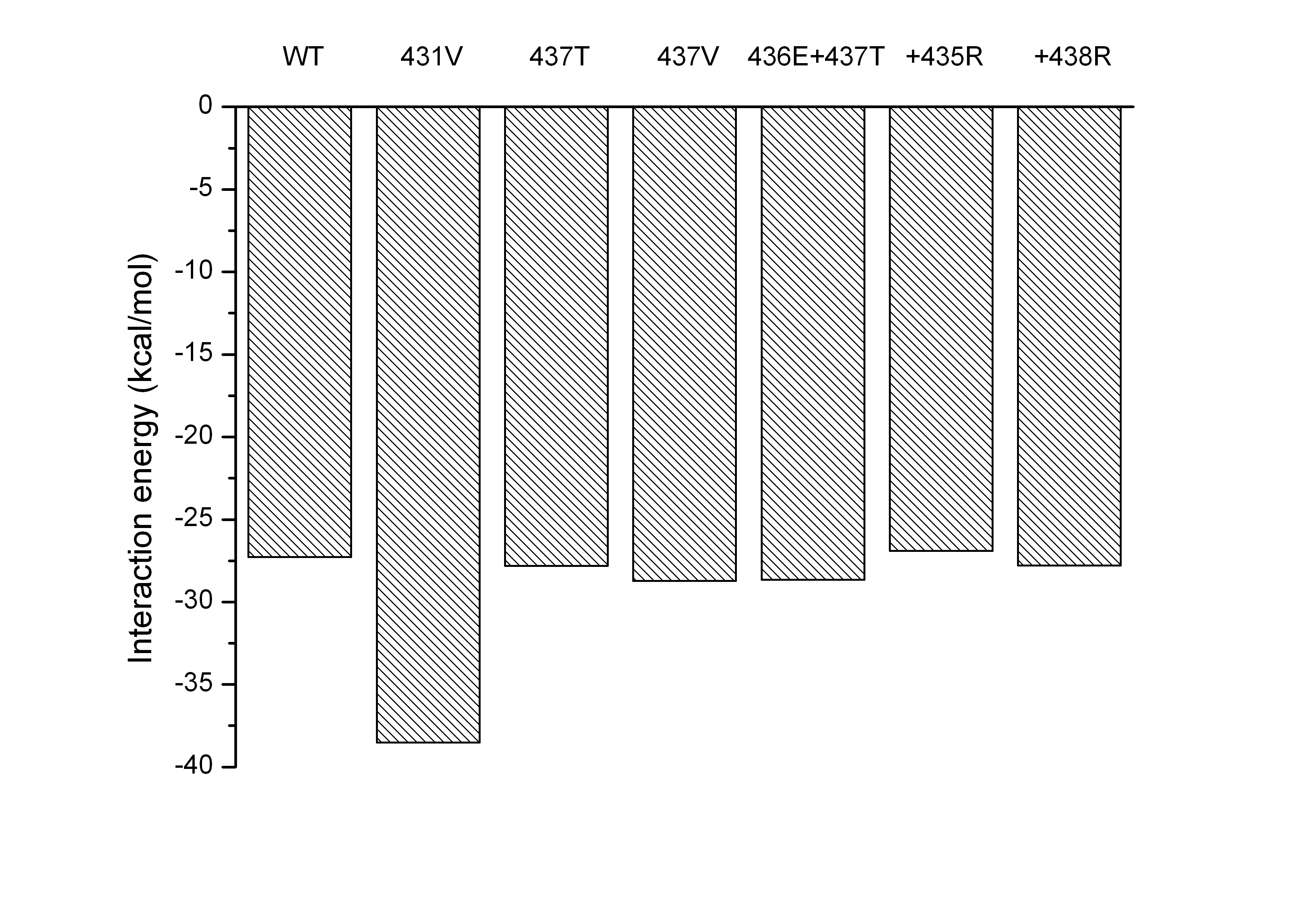

Supplement: Additional file 4 — Interaction energy between PR and substrate variants for tridecameric peptides and P3-P3' residues. [file 1742-4690-9-29-S4.DOC]
