# Supplementary material for: Modulation of HIV-1 Gag NC/p1 cleavage efficiency affects protease inhibitor resistance and viral replicative capacity
Source: Retrovirology. 2012 Apr 1;9:29. doi: 10.1186/1742-4690-9-29 (PMC3349524; doi:10.1186/1742-4690-9-29)
Supplement: Additional file 5 — Molecular modeling and simulations of PR-substrate complexes. [file 1742-4690-9-29-S5.DOC]

**Additional file 5. Molecular modeling and simulations of PR-substrate complexes**

The seven PR/NC-p1 complexes were set up for calculations, relaxed and solvated in a box of water molecules. The starting geometry was taken from the crystal structure of inactive (D25N) wild-type HIV-1 PR complexed with the NC-p1 substrate peptide (PDB code: 2FNS) [29]. The A431V substrate change within the active site was modeled by an automatic rotamer selection in Insight II package (InsightII; Accelrys Software Inc., 2000). For substrate residues outside the well-defined S3-S3’ PR pockets no experimental electron densities had been observed. We thus modeled the flanking amino acids on both unprimed and primed sides to yield the tridecapeptides studied experimentally. The residues were added so as not to clash with van der Waals surface of PR visualized with Insight II nor with the crystallographic waters that were all included in the model.

All other transformations and calculations were done using modules of AMBER 8.0 program suite (Case, D. A., T. A. Darden, T. E. Cheatham III, C. L. Simmerling, J. Wang, R. E. Duke, R. Luo, K. M. Merz, B. Wang, D. A. Pearlman, M. Crowley, S. Brozell, V. Tsui, H. Gohlke, J. Mongan, V. Hornak, G. Cui, P. Beroza, C. Schafmeister, J. W. Caldwell, W. S. Ross, W, and P. A. Kollman. 2004. AMBER 8, University of California, San Francisco).

Hydrogen atoms were added and the tridecapeptides were capped at the N- and C-termini by acetyl and N-methyl amide groups, respectively. Side chains of acidic (Asp, Glu) and basic (Lys, Arg, His) amino acids, were treated as ionic as well as the N- and C-termini of the PR. The only exception was the protonated “inner” Oδ2 oxygen atom of the catalytic Asp25 to reflect its experimentally measured pKa of ~ 6 [38]. Force-field parameters for both the protease and the substrates were taken from the ff03 set [39].

The relaxation of the structures consisted in several steps: i) 1000 cycles of minimization and 50 ps of high-temperature (500 K) dynamics of hydrogen atoms, ii) 10 000 cycles of minimization, 20ps warming to 300 K, 100ps equilibration at 300K and 500ps of high-temperature (500K) dynamics of the flanking substrate residues. The added residues were further minimized using generalized Born implicit solvent model, followed by an optimization of the whole complexes [40]. TIP3P water molecules were added in the Leap module and a three-step molecular dynamics war run: gradual warming to 300 K over 50 ps, ii) equilibration at 300 K for 200 ps and iii) production run at 300 K for 1 ns. Snapshots of the trajectory were saved every 1 ps. Vibrations of hydrogen atoms were constrained using the SHAKE algorithm. Time step of 1 fs was used for the high temperature dynamics and of 2 [[1]](#footnote-2)fs for 300K dynamics. Frames were saved every 1 [[2]](#footnote-3)ps. Cutoff for nonbonded interactions was set to 9 Å. Molecular dynamics was run in NVT (isothermal-isochoric) ensemble for the vacuum preparation and in NPT (isothermal-isobaric) ensemble for the solvated runs. Temperature was controlled using Langevin dynamics with collision frequency of 2 ps^-1^. Pressure was regulated via weak-coupling algorithm with a relaxation time of 2 ps.

1.  [↑](#footnote-ref-2)
2. [↑](#footnote-ref-3)
